# Effect of Rearing System and Seasonality on Fatty Acid Composition in *Longissimus lumborum* Muscle of Cattle in Eastern Amazon, Brazil

**DOI:** 10.1155/vmi/2468294

**Published:** 2026-08-25

**Authors:** Andrea Viana da Cruz, Jamile Andréa Rodrigues da Silva, André Guimarães Maciel e Silva, Susana Paula Alves, Cristina Fernandes Xavier, Rui José Branquinho Bessa, Ana Paula Damasceno Ferreira, Adriny dos Santos Miranda Lobato, Thomaz Cyro Guimarães de Carvalho Rodrigues, Vanessa Vieira Lourenço Costa, Antônio Marcos Quadros Cunha, Elton Alex Corrêa da Silva, Leonel António Joaquim, Laíza de Kássia Mendes da Conceição, Mariana Jucá Moraes, Welligton Conceição da Silva, José de Brito Lourenço Júnior

**Affiliations:** ^1^ Postgraduate Program in Animal Science (PPGCAN), Institute of Veterinary Medicine, Federal University of Para (UFPA), Castanhal 68746-360, Brazil, ufpa.br; ^2^ Institute of Animal Health and Production, Federal Rural University of the Amazon (UFRA), Belém 66077-830, Brazil; ^3^ Interdisciplinary Research Center in Animal Health, Faculty of Veterinary Medicine, University of Lisbon (CIISA/FMV), Lisbon 1300-477, Portugal; ^4^ Associated Laboratory for Animal and Veterinary Science (AL4Animals), Faculty of Veterinary Medicine, University of Lisbon, Lisbon 1300-477, Portugal, ulisboa.pt; ^5^ Health Science Institute, Federal University of Para, Belém CEP 66075-110, Brazil, ufpa.br; ^6^ Faculty of Agronomy, Federal University of Para, Cametá 68400-000, Brazil, ufpa.br; ^7^ Angonia Zootechnical Station (EZA), Agricultural Research Institute of Mozambique (IIAM), Angónia Tete, 2306, Mozambique

**Keywords:** beef, *Bos indicus*, human health, nutritional indexes, saturated fatty acids, unsaturated fatty acids

## Abstract

This study aimed to investigate the relationship between rearing systems and seasonality (rainy and dry seasons) on the fatty acid (FA) composition in cattle. Samples of the *Longissimus lumborum* muscle were obtained from carcasses of 96 castrated Nelore cattle. The samples were lyophilized, and the FA composition was determined by the direct transesterification method and analyzed by gas chromatography. The rearing systems comprise two native pastures (extensive grazing), one cultivated pasture (intensive grazing) and one feedlot. Data were analyzed using generalized linear models, including the fixed effects of production system, season, and their interaction. Regarding polyunsaturated fatty acids (PUFAs), linoleic acid (C18:2n‐6), arachidonic acid (C20:4n‐6), and α‐linolenic acid (C18:3n‐3) were the most abundant in all systems. C18:2n‐6, C18:3n‐3, and C20:4n‐6 were higher in pasture systems than in the feedlot, especially in intensive grazing during the rainy season. In the dry season, the scenario was reversed; C18:2n‐6 and C20:4n‐6 showed a higher concentration in the feedlot system compared to pasture systems. The study demonstrated that pasture production, such as traditional extensive systems, promoted a better ratio of n‐6/n‐3, contributing to a healthier lipid composition in the meat, when compared to the feedlot. Although the feedlot system has a lower concentration of saturated fatty acids (SFAs), the analysis shows that the extensive system offers a more balanced and healthy FA composition.

## 1. Introduction

Beef is an important source of food and energy. The Zebu breeds are the basis of Brazilian cattle breeding, where more than 80% of the cattle herd is of the Nelore breed (*Bos indicus*) and its crossbreds, raised mainly in a pasture system, with seasonal variations and in confinement systems [1]. Beef has a high bioavailability of nutrients, which are essential for the proper functioning of the human body [2, 3], and has a prominent place in most existing cultures, being considered the basis of daily meals in the most different places in the world [3, 4].

In ruminants, meat presents complexity in its fatty acid (FA) profile, when compared to that of other animal species, such as poultry or pigs, justified by the action of ruminal lipid metabolism of unsaturated FA from the diet. The lipid content in beef can be variable, as it depends on different intramuscular fat depositions, which is affected by intrinsic factors [5] such as genotype, cell differentiation, maturation state or type of muscle, and extrinsic factors, such as rearing system, mainly by feeding the animals on pasture, concentrate, or different proportions of both, which influences the nutritional value of meat lipids [6, 7]. Rumen biohydrogenation is a critical step in dietary FA metabolism and is largely responsible for FA diversity in meat [7].

Ruminant meat is characterized by containing variable amounts of saturated and trans FA, including a complex profile in 18:1 trans isomers and conjugated linoleic acid (CLA) isomers, such as CLA‐*cis*‐9, *trans*‐11, which is beneficial to human health [5]. Current nutritional recommendations encourage increased intake of n‐3 PUFA, in particular, eicosapentaenoic acid (EPA, C20:5n‐3) and docosahexaenoic acid (DHA, C22:6n‐3) [8–10]. Dietary n‐3 PUFAs have anti‐inflammatory, immunomodulatory, and anticancer potential [11, 12] and can prevent some diseases, such as cystic fibrosis, Type II diabetes, dysmenorrhea, schizophrenia, and cardiovascular diseases [11].

Considering the relationship between the FA ingested by the animals and those that deviate intact from the rumen and will be deposited in the animal tissues, great attention has been paid to dietary sources capable of modifying the FA composition of animal derived‐foods [12–14]. It is hypothesized that the FA profile of beef cattle is influenced by Eastern Amazonian production systems and seasonal variations. Therefore, the objective of this study was to characterize the FA profile of the *Longissimus lumboru*m muscle of cattle from rearing systems in the Eastern Amazon, Brazil.

## 2. Material and Methods

### 2.1. Ethical Considerations

This work was exempted from formal analysis by the Ethics Committee on the Use of Animals of the Federal Rural University of the Amazon, Belém, Pará, Brazil, through Protocol No. 1928240123, dated 08.01.2023, for the use of slaughtered animals.

### 2.2. Production Systems

**FIGURE 1:**
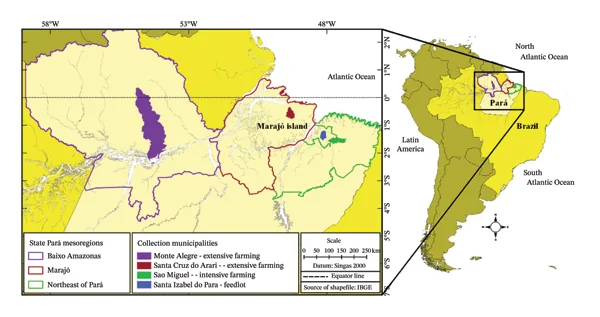
Location of the farm sampled.

Four beef cattle farms were selected (Figure 1), three of which were pasture rearing systems and one feedlot as follows:

1. Native pasture of partially flooded lands, in Santa Cruz do Arari (mesoregion of Marajó, Pará); cattle were raised in continuous grazing, composed of *Echinochloa polystachya*, *Hymenachne amplexicaulis*, *Leersia hexandra*, *Luziola spruceana*, *Oryza latifolia*, *Oryza perennis*, *Panicum coloratum*, *Paspalum fasciculatum*, and *Paspalum repens*, and water intake ad libitum. The average weight at slaughter was 410 kg at the age of 36 months.
2. Native pasture of low and upland, called “covered fields,” in Monte Alegre (Lower Amazon Mesoregion, Pará); cattle were also raised in continuous grazing, composed of *Cynodon dactylon*, *E. polystachya*, *H. amplexicaulis*, *L. hexandra*, *L. spruceana*, *O. latifolia*, *O. perennis*, *P. coloratum*, *P. fasciculatum,* and *P. repens*, and slaughtered with an average weight of 450 kg at 36 months old.
3. Cultivated pasture on the mainland, in São Miguel do Guamá (Northeast Pará Mesoregion, Pará); cattle were raised in intensive rotational grazing, in a total area of 160 ha in paddocks formed by *Panicum Maximum cv.* The entry and exit of the animals were determined by the height of the forage; 20 kg of palm oil concentrate (*Elaeis guineensis*) and a protein core per animal/day were also provided. The intake of clean, fresh water was ad libitum. The average weight at slaughter was 550 kg at 24 months.
4. Feedlot system, in Santa Izabel do Pará (Metropolitan Mesoregion of Belém, Pará); cattle were kept in a total area of 943 ha for 96 days, with an initial weight of 464 kg and slaughtered at 628.7 kg. The average daily gain was 1.635 kg. The average carcass weight of the slaughter was 341 kg with 55.81% yield. The cattle were fed for 135 days, consisting of soybean meal, barley, *Mombasa grass silage*, corn meal, cassava hulls, urea, and high‐performance core.

### 2.3. Selected Animals and Specimens

**FIGURE 2:**
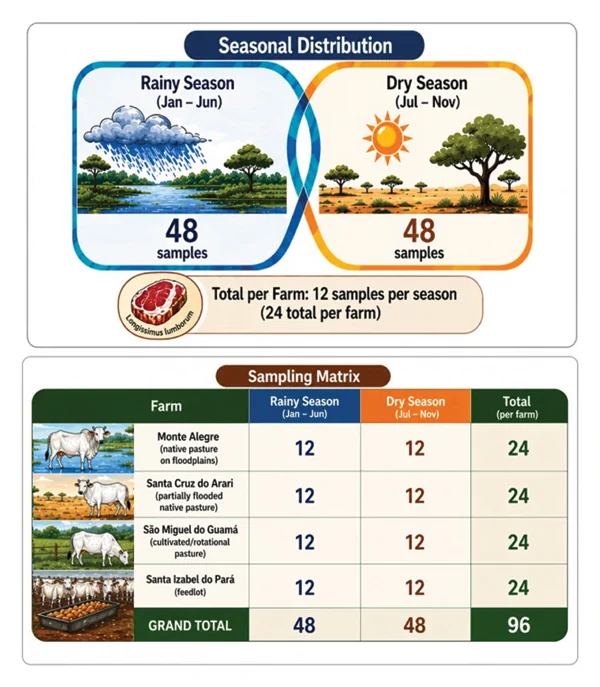
Sample size collection per system and per season.

Muscle samples of *Longissimus lumborum* were collected from 96 castrated Nelore cattle, at two times of the year (Figure 2), 48 samples in the rainy season (January to June) and the second 48 samples in the dry season (July to November), 12 samples for each farm in both seasons [15]. Samples were collected at a commercial slaughterhouse, in the amount of 100 g per animal, at a depth of 5 cm, then packed, vacuum sealed, and stored at −80°C for subsequent analysis.

### 2.4. Preparation of Muscle Tissue Samples

Muscle samples were lyophilized for 48 h at 400 mmHg, without interruption, in a lyophilizer (LIOTOP, L101), at the Animal Nutrition Laboratory of the Federal Rural University of the Amazon, Belém, Pará, Brazil, then crushed, packaged, and sent to the Interdisciplinary Research Centre in Animal Health, Faculty of Veterinary Medicine, University of Lisbon, Portugal, for FA analysis.

### 2.5. Analysis of FAs

Muscle FAs were analyzed as FA methyl ester (FAME) derivatives, prepared by direct transesterification of muscle samples according to Alves et al. [16]. FA analyses in the diet samples were performed according to the method of direct transesterification of food adapted from Sukhija and Palmquist [17]. Table 1 shows the composition of the ingredients of the diets fed to the animals belonging to the rearing systems studied.

**TABLE 1:** Chemical composition and fatty acid profile of the diet during the more and less rainy seasons.

Chemical composition (%DM)	Extensive grazing				Intensive grazing				Feedlot	
	Santa Cruz		Monte Alegre		Palm sludge		São Miguel		Santa Izabel	
	RS	DS	RS	DS	RS	DS	RS	DS	RS	DS
Dry matter	39.14	20.2	21.33	19.56	43.45	27.84	29.75	42.94	54.57	42.37
Organic matter	91.92	93.42	90.45	91.24	74.99	88.56	94.53	94.16	89.92	90.92
Crude protein	11.75	5.25	7.69	8.45	29.67	13.61	8.17	10.61	12.72	27.96
Ether extract	2.23	1.57	1.59	1.27	6.32	13.54	1.50	1.45	3.37	4.03
Neutral detergent fiber	73.64	74.88	73.4	71.54	35.15	56.08	74.27	70.41	30.72	36.07
Acid detergent fiber	25.23	68.71	27.53	65.5	7.20	47.27	26.83	63.87	11.26	22.05
Total digestible nutrients	48.99	29.27	47.51	32.28	54.37	49.34	46.9	33.81	79.55	67.71
Nonfiber carbohydrates	4.31	6.05	7.77	9.98	3.85	5.34	10.59	11.69	43.11	41.87
Ash	8.08	9.77	9.66	8.76	25.01	11.44	15.24	5.84	10.08	29.16

*Fatty acids (g/100 g total FA)*										
C14:0	0.94	0.43	1.50	0.92	3.80	0.63	0.89	0.75	0.51	0.81
C16:0	34.65	26.91	33.81	27.37	26.95	28.36	26.75	23.40	22.94	21.95
C18:0	4.26	3.44	5.67	3.05	4.07	3.33	2.42	2.45	3.30	3.65
C18:1*cis*‐9	2.45	6.07	4.87	3.81	50.54	52.92	2.77	3.14	23.21	20.41
C18:1*cis*‐11	1.39	0.59	1.74	0.43	0.08	0.95	0.50	0.42	1.02	1.02
C18:2n‐6	16.85	30.56	10.38	21.85	12.83	12.28	21.83	22.86	44.00	43.63
C20:0	0.82	1.05	1.43	0.89	0.34	0.30	0.47	0.45	0.44	1.64
C18:3n‐3	35.32	27.91	35.71	39.08	1.01	0.95	42.32	44.94	3.64	4.81
C22:0	1.07	1.29	1.60	0.91	0.14	0.08	0.78	0.82	0.44	0.89
C24:0	2.24	1.75	3.29	1.69	0.24	0.20	1.26	0.77	0.51	1.18

Abbreviations: DS, dry season; RS, rainy season.

### 2.6. Gas Chromatography Analysis

FAME was analyzed by gas chromatography (GC) with a flame ionization detector (FID) using the Shimadzu 2010Plus instrument (Shimadzu, Kyoto, Japan), equipped with a fused silica capillary column (SP‐2560, 100 m × 0.25 mm id × 0.20 μm film thickness, Supelco, Bellefonte, PA, USA). During the analysis, the injector and detector were kept at 220°C and 250°C, respectively. Helium was used as a carrier gas at a constant flow rate of 1 mL/min, and 1 μL of sample was injected in the split mode of 1:50.

The oven temperature was programmed to start at 50°C (maintained for 1 min), then increased by 50°C/minute, up to 150°C (maintained for 20 min), increased by 1°C/minute, up to 190°C (maintained for 1 min), and finally increased to 2°C/minute, up to 220°C, where it was maintained for 30 min. The identification of FA was made by comparison with commercial standards (FAME mix 37 components, Supelco Inc., Bellefonte, PA, USA). In addition, FA identifications were confirmed by mass spectrometry (MS) coupled to GC using a Shimadzu GC‐MS QP2010 Plus equipment (Shimadzu, Kyoto, Japan). The conditions of the GC, including the capillary column, were similar to those used in the GC‐FID analysis and the conditions of the MS were as follows: temperature of the ionization source, 200°C; interface temperature, 220°C; ionization energy, 70 eV; and scan interval, 0.5 s. For the identification of FAME and dimethyl acetal (DMA), an in‐house mass spectral library and the NIST 08 MS library were used. FA and DMA were expressed as a percentage of total identified peaks in the chromatogram (g/100 g total FAME + DMA). Total lipids (TLs) were calculated using lipid conversion factors proposed by Weihrauch et al. [3] from total FA quantified using the internal standard technique.

### 2.7. Diet Collection and Chemical Analysis

Pasture samples from each system evaluated were collected at five different points of 1 m^2^, later homogenized, and a sample of 1 kg was isolated for analysis at the Animal Nutrition Laboratory of the Federal University of Pará/Castanhal *Campus*. In addition, diet samples were obtained from animals raised in a feedlot, once in each seasonal period.

The samples were dried in an oven at 60°C for 24–72 h, ground in a Willey mill, using a 1‐mm sieve, and then analyzed for crude protein (INCT‐CA N001/1) [18], ash (INCT‐CA M‐001/1), ether extract (INCT‐CA G‐004/1), neutral detergent fiber (NDF), and acid detergent fiber (ADF) (INCT‐CA F‐001/1, INCT‐CA F‐002/1, INCT‐AC F‐003/1, and INCT‐AC F‐004/1), with subsequent corrections for protein and ash content [19]. Nonfiber carbohydrates were evaluated by the Sniffen method [20], and total digestible nutrients (TDNs) were calculated by the Clemson University equation: TDN = 93.59 − (ADF × 0.936).

### 2.8. Nutritional Indices of the Lipid Fraction

The enzymatic activity indices of Δ9‐desaturase for C18 FA and C16 FA and elongase were estimated according to Malau‐Aduli et al. [21], using mathematical indices

(1) Δ916100−desaturase =C1619:cis−C1619160:cis−+C:,

(2) Δ918100−desaturase =C1819:cis−C1819180:cis−+C:,

(3) elongase=100C1801819:+C:cis−C16016191801819:+C:cis−+C:+C:cis−.

The atherogenicity index (AI), thrombogenicity index (TI), and monounsaturated fatty acids (MUFAs) were estimated according to Ulbricht and Southgate [22]:

(4) AI=C1204160:+C140:+C:ΣPUFA n−63+ΣPUFA n−+ΣMUFA,

(5) TI=140:+160:+180:0.5×ΣMUFA+0.56×ΣPUFA n−+33×ΣPUFAn−+ΣPUFA n−3 ΣPUFA n−6.

The hypocholesterolemic/hypercholesterolemic ratio (*h/H*) was calculated using the equation previously proposed by Santos‐Silva et al. [4]:

(6) hH=C181918261833203620462053224622532263:n−+C:n−+C:n−+C:n−+C:n−+C:n−+C:n−+C:n−+C:n−C140160:+C:.

### 2.9. Statistical Analysis

The experiment was conducted using a completely randomized design in a 4 × 2 factorial arrangement, comprising four production systems (two extensive pasture‐based systems, one intensive pasture‐based system and one feedlot system) and two seasons (rainy and dry). Data were analyzed using generalized linear models, including the fixed effects of production system, season, and their interaction. A Gaussian distribution with an identity link function was assumed, allowing for heterogeneous variances among production systems.

Differences among fixed effects and their interaction were assessed using the Type III Wald chi‐square test, and multiple comparisons were performed using Sidak‐adjusted pairwise tests (α = 0.05). When a significant interaction was detected, comparisons were conducted among interaction levels. In the absence of a significant interaction, differences were evaluated based on the main effects of the season and farm.

Due to the large number of FAs analyzed, we reported only those FAs with values exceeding 1% in at least one treatment (the complete dataset is provided in Table S1). Results are reported as estimated marginal means (EMMs) ± standard error. All analyses were performed in R Version 4.5.2 (R Core Team, 2025), using the glmmTMB package for generalized linear modeling [23], the car package for Type III Wald tests [24], and the emmeans package for estimation of EMMs and multiple comparisons [25].

As a complement, the multivariate principal component analysis (PCA) technique was performed, using the first two principal components of greatest relevance. To carry out the PCA, initially the matrix of residues referring to the FA that represented more than 1% in at least one treatment was transformed into the “log + 1” scale and then standardized. The standardization was performed using the scale function. The PCA was performed using the factor Miner package, and the visualization of the biplot using the factoextra package [26].

## 3. Results

The highest concentrations of saturated fatty acids (SFAs) occurred in the extensive grazing system (locality of Monte Alegre in the rainy season and Santa Cruz in both seasons). On the other hand, the lowest concentrations of SFAs occurred in the feedlot system in the dry season and in the intensive grazing system in the rainy season. Regarding MUFAs, the highest concentration was recorded in the feedlot system and the lowest concentration in intensive grazing, both in the rainy season. Concerning PUFAs, the highest concentrations were measured in the feedlot in the dry season and intensive grazing in the rainy season, and the lowest concentrations occurred in extensive grazing (Monte Alegre in both seasons and Santa Cruz in the dry season). For DMA, the highest concentration was recorded in intensive grazing in the rainy season and the lowest in extensive grazing (Santa Cruz) and intensive grazing, both in the dry season.

The FAs with the highest concentrations were oleic acid (C18:1c9), palmitic acid (C16:0), stearic acid (C18:0), and C18:2n‐6, corresponding to approximately 80% of the total FAs in all rearing systems and in both seasons (Table 2). The high proportion of C16:0 and C18:0 in meat contributed to the higher proportion of SFAs (40.1%–50.1% of total FAs) compared to the other classes of FAs. Only in the feedlot system in the rainy season, the proportion of MUFAs exceeded the proportion of SFAs (46.0 vs 44.3% of total FAs).

**TABLE 2:** Mean concentrations (% total identified peaks) and estimated marginal means ± standard errors of fatty acids and dimethyl acetals (DMAs) in the *Longissimus lumborum* muscle of Nelore cattle.

	Extensive grazing				São Miguel (intensive grazing)		Feedlot (Santa Izabel)		Systems	Season	Systems x season
	Santa Cruz		Monte Alegre								
	RS	DS	RS	DS	RS	DS	RS	DS			
*Saturated and branched chain*											
C14:0	1.89 ± 0.19^bA^	1.99 ± 0.19^bA^	2.86 ± 0.12^aA^	2.33 ± 0.12^bB^	1.58 ± 0.25^bB^	3.06 ± 0.25^aA^	3.15 ± 0.21^aA^	1.75 ± 0.21^bB^	*p* < 0.001	0.536	*p* < 0.001
C16:0	21.06 ± 0.87^cA^	22.34 ± 0.87^aA^	24.99 ± 0.48^aA^	23.99 ± 0.48^aA^	21.68 ± 0.66^bcB^	23.95 ± 0.66^aA^	24.28 ± 0.75^abA^	18.51 ± 0.75^bB^	*p* < 0.001	0.107	*p* < 0.001
C17:0	1.13 ± 0.04^aA^	1.24 ± 0.04^aA^	1.08 ± 0.05^aA^	1.10 ± 0.05^aA^	0.45 ± 0.04^cB^	0.71 ± 0.04^bA^	0.77 ± 0.05^bA^	0.80 ± 0.05^bA^	*p* < 0.001	0.001	0.016
C18:0	24.96 ± 0.98^a^	23.39 ± 0.98^a^	20.40 ± 0.85^b^	20.13 ± 0.85^b^	17.39 ± 0.63^c^	18.87 ± 0.63^c^	15.42 ± 0.97^c^	18.46 ± 0.97^c^	*p* < 0.001	0.275	0.073

*Monounsaturated FA*											
C16:1c9	1.57 ± 0.09^bA^	1.69 ± 0.09^bA^	1.90 ± 0.10^bA^	1.83 ± 0.10^abA^	1.60 ± 0.11^bB^	2.13 ± 0.11^aA^	2.78 ± 0.16^aA^	1.83 ± 0.16^abB^	*p* < 0.001	0.267	*p* < 0.001
C18:1c9	30.81 ± 0.73^bcA^	32.67 ± 0.73^abA^	34.73 ± 1.32^abA^	35.73 ± 1.32^aA^	28.65 ± 0.77^cB^	32.35 ± 0.77^abA^	37.15 ± 1.24^aA^	29.93 ± 1.24^bB^	*p* < 0.001	0.828	*p* < 0.001
C18:1t11	2.06 ± 0.14^aB^	2.50 ± 0.14^aA^	1.50 ± 0.12^bA^	1.56 ± 0.12^bA^	1.13 ± 0.10^bcB^	1.48 ± 0.10^bA^	0.89 ± 0.11^cA^	0.43 ± 0.11^cB^	*p* < 0.001	0.269	*p* < 0.001
C18:1c11	0.87 ± 0.06^bA^	0.86 ± 0.06^cA^	0.96 ± 0.06^abA^	1.12 ± 0.06^bA^	1.16 ± 0.06^aA^	1.16 ± 0.06^bA^	1.18 ± 0.08^aB^	1.89 ± 0.08^aA^	*p* < 0.001	*p* < 0.001	*p* < 0.001

*Polyunsaturated FA*											
C18:2n‐6	3.54 ± 0.42^bA^	2.81 ± 0.42^cA^	2.10 ± 0.21^cA^	2.49 ± 0.21^cA^	12.09 ± 0.82^aA^	5.80 ± 0.82^bB^	5.28 ± 0.97^bB^	13.35 ± 0.97^aA^	*p* < 0.001	0.451	*p* < 0.001
C18:3n‐3	1.02 ± 0.09^bA^	0.79 ± 0.09^aA^	0.75 ± 0.06^bcA^	0.80 ± 0.06^aA^	1.70 ± 0.10^aA^	0.68 ± 0.10^aB^	0.46 ± 0.13^cB^	0.89 ± 0.13^aA^	*p* < 0.001	0.005	*p* < 0.001
C20:4n‐6	1.33 ± 0.13^bA^	0.92 ± 0.13^bB^	0.60 ± 0.08^cA^	0.72 ± 0.08^bA^	2.62 ± 0.24^aA^	1.11 ± 0.24^bB^	0.94 ± 0.22^bcB^	2.61 ± 0.22^aA^	*p* < 0.001	0.792	*p* < 0.001
C22:5n‐3	0.86 ± 0.07^bA^	0.64 ± 0.07^bB^	0.41 ± 0.05^cA^	0.47 ± 0.05^bA^	1.23 ± 0.12^aA^	0.67 ± 0.12^bB^	0.44 ± 0.12^cB^	1.18 ± 0.12^aA^	*p* < 0.001	0.903	*p* < 0.001

*Dimethyl acetals (DMAs)*											
DMA‐C16:0	0.43 ± 0.07^bA^	0.17 ± 0.07^bB^	0.45 ± 0.07^bA^	0.28 ± 0.07^abA^	1.13 ± 0.21^aA^	0.23 ± 0.21^abB^	0.19 ± 0.07^bB^	0.44 ± 0.07^aA^	0.088	0.002	*p* < 0.001

*Note:* When a significant interaction was detected, different lowercase letters within the same row indicate significant differences among rearing systems within each season, whereas different uppercase letters within the same row indicate significant differences between seasons within each rearing system (Sidak adjustment, *p* < 0.05). When the interaction was not significant, lowercase and uppercase letters indicate differences among the main effects evaluated separately (Sidak adjustment, *p* < 0.05). *p* values for the rearing system, season, and their interaction were obtained using Type III Wald chi‐square tests.

Abbreviations: DR = rainy season; DS = dry season.

For C14:0 FA, a significant interaction was observed between the rearing system and season (*p* < 0.001). During the rainy season, the highest concentrations were observed in the Monte Alegre and feedlot systems (2.86% and 3.15%, respectively; *p* < 0.05), whereas the São Miguel system exhibited the lowest value (1.58%). In the dry season, the São Miguel system reached the highest mean (3.06%). Regarding the seasonal effect, the transition to the dry period significantly decreased C14:0 levels in the São Miguel and Monte Alegre systems, but promoted a significant increase in the feedlot system (*p* < 0.05).

The C16:0 FA also showed a significant interaction effect (*p* < 0.001). During the rainy season, the Monte Alegre system and the feedlot obtained the highest proportions (24.99% and 24.28%, respectively), while the lowest value was recorded in the Santa Cruz system (21.06%). In the dry season, the highest proportions were concentrated in the pasture‐based systems, whereas the lowest content was observed in the feedlot. The seasonal effect was significant for the cultivated pasture system, which increased from 21.68% in the rainy season to 23.95% in the dry season, and for the feedlot, it decreased from 24.28% to 18.51% during the same period.

For C17:0, significant effects were verified for the rearing system (*p* < 0.001), season (*p* = 0.001), and their interaction (*p* = 0.016). In the rainy season, contents in the extensive systems (Santa Cruz and São Miguel) were higher than those in the feedlot (*p* < 0.05). In the dry season, the Santa Cruz system maintained the highest mean (1.24%). Between seasons, only the São Miguel system underwent a significant change, showing an elevation of C17:0 in the dry season (0.71%) compared to the rainy season (0.45%).

The content of C18:0 FA was not influenced by the season (*p* = 0.275) or by the system × season interaction (*p* = 0.073). A significant main effect was found only for the rearing system (*p* < 0.001), in which the highest mean contents occurred consistently in the Santa Cruz system (24.96% and 23.39%), followed by Monte Alegre (20.40% and 20.13%), with the lowest proportions clustered in the São Miguel and feedlot systems.

The compound DMA‐C16:0 showed a significant interaction (*p* < 0.001) and a significant main effect of the season (*p* = 0.002), with no isolated effect of the system (*p* = 0.088). In the rainy season, the cultivated pasture group recorded the highest value (1.13%), while the other systems were statistically similar and lower (*p* < 0.05). In the dry season, the feedlot obtained the highest mean (0.44%). Between seasons, the transition to the dry period caused a drastic decline in DMA‐C16:0 in the Santa Cruz and cultivated pasture systems, while prompting a significant increase in the feedlot (*p* < 0.05).

Among MUFAs, the highest proportions were found in C18:1*c*9, vaccenic acid (C18:1*t*11), and palmitoleic acid (C16:1*c*9). The C16:1c9 FA exhibited a significant interaction between systems and seasons (*p* < 0.001). In the rainy season, the feedlot obtained the highest proportion (2.78%), outperforming the other treatments (*p* < 0.05). In the dry season, the São Miguel and Monte Alegre systems showed the highest values (2.13% and 1.83%, respectively). In the seasonal comparison, the São Miguel system increased C16:1c9 content during the dry period, whereas the feedlot showed a significant reduction in the same season (1.83%).

For C18:1c9, a significant interaction was recorded (*p* < 0.001). In the rainy season, the feedlot and the Monte Alegre system reached the highest contents (37.15% and 34.73%), while the São Miguel system obtained the lowest mean (28.65%). In the dry season, the highest value was observed in the Monte Alegre system (35.73%) and the lowest in the feedlot (29.93%). Between seasons, the São Miguel system registered an increase in contents during the dry period, while the feedlot showed a significant decrease (*p* < 0.05).

The C18:1t11 isomer was influenced by the system × season interaction (*p* < 0.001). In both seasons, the Santa Cruz system provided the highest concentrations of this FA (2.06% in the rainy season and 2.50% in the dry season; *p* < 0.05). Evaluating the internal seasonal effect, the Santa Cruz and São Miguel systems showed a significant increase in contents during the dry season, whereas the feedlot underwent a reduction in the dry period (0.43%).

For C18:1c11 FA, all main effects and their interaction were significant (*p* < 0.001). In the rainy season, the highest means were detected in the São Miguel and feedlot systems (1.16% and 1.18%). In the dry season, the feedlot stood out with the highest isolated value among all treatments (1.89%; *p* < 0.05). In the comparison between periods, the transition from the rainy to the dry season elevated C18:1c11 content only in the feedlot, remaining unchanged in the other systems.

Regarding PUFAs, C18:2n‐6, C20:4n‐6, and C18:3n‐3 were the most abundant PUFAs in all systems. The C18:2n‐6 FA exhibited a significant interaction (*p* < 0.001). In the rainy season, the cultivated pasture system obtained the highest concentration (12.09%), followed by the feedlot (5.28%). In the dry season, the scenario was reversed: the feedlot recorded the highest value (13.35%), followed by the cultivated pasture (5.80%). The native pasture systems (Santa Cruz and Monte Alegre) maintained the lowest contents in both seasons. Internally, the cultivated pasture reduced its C18:2n‐6 contents in the dry season, while the feedlot demonstrated a significant elevation during the same period (*p* < 0.05).

For C18:3n‐3, a significant interaction effect was verified (*p* < 0.001). In the rainy season, the cultivated pasture presented the highest mean (1.70%) and the feedlot the lowest (0.46%). In the dry season, however, the proportions remained similar among all systems (*p* > 0.05). Seasonal analysis indicated that C18:3n‐3 content decreased during the dry season within the cultivated pasture system, but increased significantly in the feedlot (*p* < 0.05).

The C20:4n‐6 FA demonstrated a similar behavior to its precursor (C18:2n‐6), with a significant interaction recorded (*p* < 0.001). In the muscle of animals from the cultivated pasture, the highest content was observed in the rainy season (2.62%) followed by a reduction in the dry season (1.11%). Conversely, the feedlot started with lower content in the rainy season (0.94%) and increased significantly in the dry season (2.61%). The native pasture systems (Santa Cruz and Monte Alegre) presented the lowest values, and a reduction in the dry season was also observed in Santa Cruz (0.92%).

For C22:5n‐3 FA, a significant interaction was observed (*p* < 0.001). In the rainy season, the highest value belonged to samples from the cultivated pasture system (1.23%) and the lowest to the Monte Alegre system (0.41%). In the dry season, the feedlot obtained the highest proportion (1.18%). The seasonal effect indicated a reduction in contents during the dry season for the Santa Cruz and São Miguel systems, contrasting with the feedlot, which significantly increased its concentration in the dry period (*p* < 0.05).

The concentration of total *n*‐6 and *n*‐3 was not affected by the season (*p* > 0.05). Meat from cultivated pasture and feedlot systems presented a higher concentration of n‐6, meat from the extensive pasture had a lower concentration, meat from the extensive pasture in Monte Alegre presented a higher proportion of *n*‐3, and meat from São Miguel and feedlot systems had a lower proportion (*p* < 0.05, Table 3).

**TABLE 3:** Estimated marginal means ± standard errors of the sum, fatty acid ratios, and nutritional indices and desaturase indices in *the Longissimus lumborum* muscle of Nelore cattle.

	Extensive grazing				São Miguel (intensive grazing)		Feedlot (Santa Izabel)		Systems	Season	Systems × season
	Santa Cruz		Monte Alegre								
	RS	DS	RS	DS	RS	DS	RS	DS			
*Partial sums (% total fatty acids)*											
SFA	49.61 ± 1.16^aA^	49.93 ± 1.11^aA^	50.05 ± 1.03^aA^	48.28 ± 1.03^aA^	41.63 ± 1.17^bB^	47.37 ± 1.17^aA^	44.25 ± 1.25^bA^	40.10 ± 1.25^bB^	*p* < 0.001	0.969	*p* < 0.001
MUFA	38.75 ± 0.81^bcB^	41.04 ± 0.78^abA^	42.06 ± 1.37^abA^	43.38 ± 1.37^aA^	35.62 ± 0.88^cB^	41.16 ± 0.88^abA^	46.00 ± 1.38^aA^	37.81 ± 1.38^bB^	*p* < 0.001	0.764	*p* < 0.001
*cis*‐MUFA	35.60 ± 0.92^bcA^	37.49 ± 0.88^aA^	39.69 ± 1.48^abA^	40.89 ± 1.48^aA^	32.85 ± 0.97^cB^	37.83 ± 0.97^aA^	43.76 ± 1.42^aA^	35.72 ± 1.42^aB^	*p* < 0.001	0.992	*p* < 0.001
*trans*‐MUFA	2.79 ± 0.16^aA^	3.20 ± 0.15^aA^	2.08 ± 0.14^bA^	2.13 ± 0.14^bA^	2.35 ± 0.19^abB^	2.91 ± 0.19^aA^	1.92 ± 0.11^bA^	1.71 ± 0.11^bA^	*p* < 0.001	0.055	0.026
n‐PUFA	7.83 ± 0.74^bA^	5.92 ± 0.71^bcA^	4.29 ± 0.42^cA^	5.05 ± 0.42^cA^	19.27 ± 1.23^aA^	9.05 ± 1.23^bB^	7.75 ± 1.52^bcB^	19.86 ± 1.52^aA^	*p* < 0.001	0.805	*p* < 0.001
n‐6 PUFA	1.27 ± 0.16^b^	1.14 ± 0.15^b^	1.41 ± 0.11^b^	1.26 ± 0.11^b^	1.73 ± 0.14^a^	1.90 ± 0.14^a^	1.78 ± 0.25^a^	2.07 ± 0.25^a^	*p* < 0.001	0.716	0.434
n‐3 PUFA	0.56 ± 0.07^bc^	0.49 ± 0.06^bc^	0.67 ± 0.06^a^	0.55 ± 0.06^a^	0.43 ± 0.06^b^	0.45 ± 0.06^b^	0.29 ± 0.04^c^	0.32 ± 0.04^c^	*p* < 0.001	0.332	0.381

*Partial sums (mg/100 g fresh meat)*											
SFA	11.63 ± 1.67^bA^	15.34 ± 1.59^aA^	25.58 ± 2.40^aA^	18.82 ± 2.40^aB^	4.84 ± 1.44^cB^	14.36 ± 1.44^aA^	13.43 ± 1.58^bA^	5.89 ± 1.58^bB^	*p* < 0.001	0.831	*p* < 0.001
TFA (mg/g DM)	80.49 ± 10.40^bB^	110.08 ± 9.96^aA^	154.04 ± 12.94^aA^	148.65 ± 12.94^aA^	34.58 ± 5.46^cB^	78.97 ± 5.46^bA^	112.88 ± 9.13^abA^	47.43 ± 9.13^cB^	*p* < 0.001	0.910	*p* < 0.001
TFA (mg/g fresh meat)	23.55 ± 3.06^bA^	30.28 ± 2.93^aA^	51.68 ± 5.16^aA^	39.26 ± 5.16^aA^	11.68 ± 2.85^cB^	29.98 ± 2.85^aA^	30.26 ± 3.36^bA^	14.02 ± 3.36^bB^	*p* < 0.001	0.730	*p* < 0.001
TL (%)	2.57 ± 0.33^bA^	3.31 ± 0.32^aA^	5.64 ± 0.56^aA^	4.29 ± 0.56^aA^	1.27 ± 0.31^cB^	3.27 ± 0.31^aA^	3.30 ± 0.37^bA^	1.53 ± 0.37^bB^	*p* < 0.001	0.731	*p* < 0.001
n‐6 PUFA	5.42 ± 0.58^bA^	4.16 ± 0.56^cA^	2.93 ± 0.31^cA^	3.53 ± 0.31^cA^	15.55 ± 1.04^aA^	7.34 ± 1.04^bB^	6.62 ± 1.27^bB^	17.04 ± 1.27^aA^	*p* < 0.001	0.537	*p* < 0.001
n‐3 PUFA	2.41 ± 0.23^bA^	1.76 ± 0.22^bB^	1.36 ± 0.11^cA^	1.52 ± 0.11^bA^	3.72 ± 0.27^aA^	1.71 ± 0.27^bB^	1.13 ± 0.31^cB^	2.82 ± 0.31^aA^	*p* < 0.001	0.240	*p* < 0.001
EPA + DHA	0.12 ± 0.02^a^	0.09 ± 0.02^a^	0.10 ± 0.01^a^	0.09 ± 0.01^a^	0.09 ± 0.02^ab^	0.09 ± 0.02^ab^	0.06 ± 0.01^b^	0.08 ± 0.01^b^	0.006	0.507	0.108
BCFA	0.55 ± 0.08^bA^	0.70 ± 0.08^abA^	1.26 ± 0.14^aA^	0.90 ± 0.14^aA^	0.12 ± 0.05^cB^	0.48 ± 0.05^bA^	0.35 ± 0.04^bA^	0.15 ± 0.04^cB^	*p* < 0.001	0.819	*p* < 0.001

*Ratios and nutritional quality indices*											
n‐6/n‐3	2.45 ± 0.33^c^	2.34 ± 0.31^c^	2.16 ± 0.07^c^	2.28 ± 0.07^c^	4.28 ± 0.30^b^	4.46 ± 0.30^b^	6.73 ± 0.51^a^	6.64 ± 0.51^a^	*p* < 0.001	0.916	0.948
*h/H*	1.77 ± 0.11^aA^	1.60 ± 0.11^bA^	1.41 ± 0.06^bA^	1.55 ± 0.06^bA^	2.11 ± 0.11^aA^	1.54 ± 0.11^bB^	1.65 ± 0.14^abB^	2.56 ± 0.14^aA^	*p* < 0.001	0.319	*p* < 0.001
AI	0.69 ± 0.05^abA^	0.71 ± 0.05^aA^	0.85 ± 0.03^aA^	0.74 ± 0.03^aB^	0.55 ± 0.04^bB^	0.79 ± 0.04^aA^	0.73 ± 0.04^aA^	0.47 ± 0.04^bB^	*p* < 0.001	0.377	*p* < 0.001
TI	1.75 ± 0.10^abA^	1.84 ± 0.09^aA^	1.90 ± 0.07^aA^	1.73 ± 0.07^aA^	1.18 ± 0.08^cB^	1.69 ± 0.08^aA^	1.52 ± 0.08^bA^	1.16 ± 0.08^bB^	*p* < 0.001	0.718	*p* < 0.001
Δ9‐desaturase 16	6.95 ± 0.37^b^	7.06 ± 0.35^b^	7.08 ± 0.32^b^	7.04 ± 0.32^b^	6.92 ± 0.45^b^	8.20 ± 0.45^b^	10.20 ± 0.48^a^	8.94 ± 0.48^a^	*p* < 0.001	0.939	0.056
Δ9‐desaturase 18	55.93 ± 1.57^cA^	58.27 ± 1.51^aA^	62.88 ± 1.77^bA^	63.74 ± 1.77^aA^	62.17 ± 1.25^bA^	63.18 ± 1.25^aA^	70.71 ± 1.88^aA^	61.63 ± 1.88^aB^	*p* < 0.001	0.290	0.004
Elongase	71.25 ± 1.05^aA^	70.02 ± 1.00^aA^	67.19 ± 0.57^bA^	68.41 ± 0.57^abA^	66.48 ± 0.60^bA^	66.30 ± 0.60^bA^	66.03 ± 0.78^bB^	70.55 ± 0.78^aA^	*p* < 0.001	0.046	0.002

*Note:* When a significant interaction was detected, different lowercase letters within the same row indicate significant differences among rearing systems within each season, whereas different uppercase letters within the same row indicate significant differences between seasons within each rearing system (Sidak adjustment, *p* < 0.05). When the interaction was not significant, lowercase and uppercase letters indicate differences among the main effects evaluated separately (Sidak adjustment, *p* < 0.05). *p* values for the rearing system, season, and their interaction were obtained using Type III Wald chi‐square tests.

Abbreviations: DR = rainy season; DS = dry season.

The proportion of cis‐MUFAs was not influenced by the season (*p* = 0.992), but was influenced by the rearing system (*p* < 0.001), where the highest mean was observed in the feedlot during the rainy season, and the lowest mean was found in São Miguel. No difference was observed in the dry season among the systems. On the other hand, the proportion of trans‐MUFAs showed a higher concentration in the extensive system in Santa Cruz during the rainy season (*p* < 0.001). In the dry season, Santa Cruz and São Miguel presented a higher concentration, while the lower concentration was observed in Monte Alegre and feedlot systems.

In fresh meat (Table 3), it was observed that the extensive grazing (Monte Alegre) had higher proportions of SFAs in the rainy season. Intensive grazing in the rainy season and the feedlot system in the dry season had less influence on SFAs (*p* < 0.001).

For total FAs (TFAs) in fresh meat, extensive grazing had a greater influence, especially in Monte Alegre in the rainy season (*p* < 0.001). For TLs, there was an influence of rearing systems (*p* < 0.001), where extensive grazing in Monte Alegre presented the highest concentration in the rainy season, while the lowest concentration was observed in São Miguel. In the dry season, regardless of the pasture‐based system, all present higher proportion compared to the feedlot system.

For total *n*‐3 PUFAs and n‐6 PUFAs, the highest concentrations were recorded in São Miguel during the rainy season, and the lowest concentration was observed in Monte Alegre (*p* < 0.001). During the dry season, extensive grazing presented the lowest concentration, whereas the feedlot system had the highest proportion.

Regarding the proportions and indices of nutritional quality (Table 3), there were no interaction of the system and season for the *n*‐6/*n*‐3 ratio (*p* = 0.948) and no effect of the season (*p* = 0.916). Differences were only observed among systems, where the feedlot system presented the highest proportion compared to pasture‐based systems. The system × season interaction was significant for *h*/*H* (*p* < 0.001). During the rainy season, the highest proportion of *h/H* was observed in the São Miguel system, and the lowest mean was recorded in extensive grazing in Monte Alegre. In the dry season, the feedlot system presented the highest *h*/*H* index compared to pasture‐based systems. Between the seasons, no difference was observed in extensive grazing systems. In the intensive grazing system, there was a reduction from the rainy to dry season, and the inverse pattern was observed in the feedlot system.

AI was influenced by the rearing system (*p* < 0.001), with the highest value recorded in the rainy season in Monte Alegre and the feedlot (0.85 and 0.73, respectively). In the dry season, the highest values were observed in the pasture‐based system compared to the feedlot. No difference was observed between seasons in Santa Cruz, whereas there was a decrease in the dry season in Monte Alegre and the feedlot and an increase in São Miguel in the dry season.

Regarding the TI, no differences were observed in the extensive grazing system between seasons, whereas in São Miguel, there was a decrease from the rainy to dry season and an increase in the feedlot (*p* < 0.001). Comparing the rearing systems, the highest value was observed in Monte Alegre and the lowest value in São Miguel, both in the rainy season. During the dry season, all pasture‐based system presented the highest value than the feedlot system (*p* < 0.001).

No system × season interaction was observed for Δ9‐desaturase 16 activity (*p* = 0.056). The highest index of Δ9‐desaturase 16 activity was observed in the feedlot, in both periods (*p* < 0.001). On the other hand, there was a system × season interaction for Δ9‐desaturase C18 activity (*p* = 0.004). No variation was observed between seasons for pasture‐based systems, whereas Δ9‐desaturase C18 activity decreased from the rainy to dry season.

Regarding the elongase activity index, different patterns were observed between seasons, with an increase from the rainy to dry season in the feedlot system and no variation in pasture‐based systems. Among the systems, Santa Cruz presented the highest value, while Monte, Alegre, São Miguel, and the feedlot had the lowest value in the rainy season (*p* < 0.001). During the dry season, Santa Cruz and the feedlot system had the highest elongase activity index, while São Miguel registered the lowest index.

**FIGURE 3:**
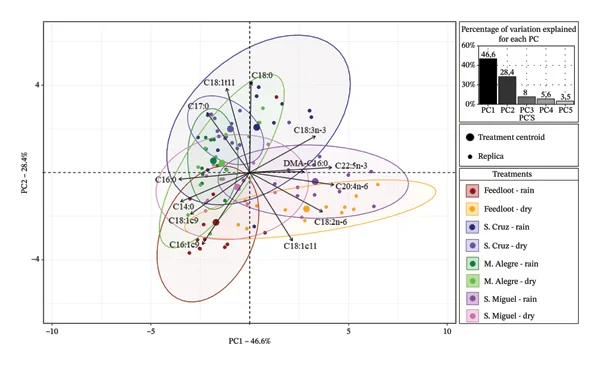
Analysis of the main components of fatty acids in the *Longissimus lumborum* muscle of cattle. PC1 = Principal Component 1; PC2 = Principal Component 2.

Figure 3 shows that the two principal components determined by PCA (PC1 and PC2) described 46.6% (PC 1) and 28.4% (PC 2) of the cumulative variance contribution rate. In the first component on the positive axis, the FAs were grouped by the long‐chain FAs C20:4n‐6, C22:5n‐3, C18:2n‐6, and C18:3n‐3 and the SFA DMA‐C16:0. On the negative axis, the samples were grouped into C16:1*c*9, C18:1*c*9, C14:0, and C16:0. In the second component, the samples were grouped in the positive axis in FA C18:0, C17:0, and C18:1*t*11, while in the negative axis, C18:1*c*11 and C16:1*c*9 were projected.

The area of influence of the ellipses indicated differences in the composition of FA among treatments. The main variation occurred in extensive grazing (Santa Cruz) in the dry season, which is located in the positive portion of PC2, and in the feedlot system in both periods, which are in the negative portion of PC2. Although PC1 represents the component with the greatest explained variation, there was no difference between the ellipses.

## 4. Discussion

This study showed that, regardless of the farming systems evaluated and the season, the FAs found in high concentrations were oleic (C18:1*c*9), palmitic (C16:0), and stearic (C18:0) acids. These FAs are commonly found in beef, so the findings of this study are in line with the literature, as previously demonstrated by Silva et al. [15] in buffalo in the Eastern Amazon, Brazil, and Lopes et al. [27] in Nellore cattle in Lavras, Minas Gerais, Brazil. The different climatic regions of each collection farm demonstrate the diversity and timing of pasture growth. These differences in climate in each region were described in the study by Lobato et al. [28].

Oleic acid is the main MUFA in meat and can have beneficial effects on human health, mainly avoiding cardiovascular problems, by lowering low‐density lipoprotein (LDL) cholesterol and increasing high‐density lipoprotein (HDL) cholesterol [29]. In addition, it can influence the stimulation of lipid synthesis from glucose, marbling, and lipogenesis from acetate [30], in addition to assisting in the expression of the enzyme Δ9‐desaturase.

Oleic acid may directly regulate the synthesis and activities of antioxidant enzymes, and its anti‐inflammatory effect may be related to the inhibition of pro‐inflammatory cytokines and the activation of anti‐inflammatory cytokines [31, 32]. In this context, as far as C18:1*c*9 is concerned, the samples analyzed are favorable to human health.

However, a high amount of C18:1*c*9 in intramuscular fat is commonly found in beef from grain‐fed cattle or in breeds that are genetically prone to fat deposition, producing meat with superior tenderness and palatability [33].

In our study, the highest deposition of C18:1*c*9 was observed in the meat of animals intensively confined during the rainy season. In a previous study with crossbred buffaloes raised extensively and intensively in the Eastern Amazon, confined animals also had elevated C18:1*c*9 in meat, reaching 39% of the total FA [15]. But interestingly, in our study, the content of C18:1*c*9 in feedlot dry‐season animals reached only 29.93% of the total FAs. The accumulation of C18:1*c*9 in the muscle derives mainly from endogenous synthesis, carried out by the enzyme Δ9‐desaturase, which introduces a cis‐9 double bond into C18:0 [34, 35]. In fact, dietary C18:1*c*9 is largely biohydrogenated in the rumen to form C18:0, which, after absorption, is available for endogenous conversion to C18:1*c*9 [36, 37]. In the present study, feedlot animals in the dry season had high C18:0 contents and lower estimated C18 Δ9‐desaturase activity indices compared to those in the rainy season, explaining the differences in the concentration of C18:1*c*9 in feedlot animals.

The Δ9‐desaturase enzyme is also responsible for the introduction of a cis‐9 double bond into C18:1*t*11, leading to the endogenous formation of rumenic acid (C18:2*c*9*t*11), an isomer of CLA, which has potential benefits for human health, including anticancer effects [6]. The lowest CLA content in meat was also detected in feedlot animals in the dry season. This may not only be related to the low activity of the Δ9‐desaturase enzyme, but mainly to the lack of substrate, i.e., the C18:1*t*11.

C18:1*t*11 is formed in the rumen from the incomplete biohydrogenation of C18:2n‐6 and C18:3n‐3 from the diet performed by rumen bacteria. However, it is known that under feedlot systems, feeding diets are high in starch and low in fiber; there is a change in the main routes of ruminal biohydrogenation of PUFA, with C18:1*t*11 being replaced by C18:1*t*10 as the main FA transformed in the rumen. This limits the escape of C18:1*t*11 from the rumen and its subsequent conversion to C18:2*c*9*t*11 in the tissues. Unlike C18:1*t*11, C18:1*t*10 can have deleterious effects on human health [16].

In our study, differences were observed between the rearing systems in the proportions of C18:1*t*11 and C18:1*t*10 in meat, with feedlot animals presenting the lowest levels of C18:1*t*11 and the highest levels of C18:1*t*10. However, the highest proportions of C18:1*t*11 and C18:2*c*9*t*11 were observed in meat from animals raised in extensive grazing (Santa Cruz) during the dry season.

Despite the extensive biohydrogenation of dietary unsaturated FAs in the rumen, some can escape and be deposited in tissues, such as C18:2n‐6 and C18:3n‐3, which are two essential FAs that must be fed to animals through the diet. C20:4n‐6 is also the dominant PUFA in meat. C18:2n‐6 is converted into C20:4n‐6, which is also a substrate to produce a wide variety of eicosanoids, some of which have inflammatory, vasoconstrictor, and/or preaggregation characteristics, such as prostaglandin, thromboxane, and leukotriene. C18:3n‐3 is converted into other health‐beneficial omega‐3 PUFAs, such as DHA and EPA. These PUFA also serve as a substrate for other eicosanoids with anti‐inflammatory/antiplatelet characteristics. Evidence also shows that increasing omega‐3 intake confers protection against cardiovascular disease, and the different sources of omega‐3, through the reduction of hepatic lipogenesis and LDL, contribute significantly to the reduction of triglycerides [38].

The concentrations of the total proportions n‐6, n‐3, and PUFA observed in this study contrasted with those observed by Silva et al. [15], who, when evaluating the *Longissimus lumborum* muscle of buffaloes in different productive systems of the Eastern Amazon, observed that the highest values were in the Marajó rearing system and lower in the feedlot. However, differences in meat PUFA ratios reflect not only differences in diets but also differences in the intramuscular fat content. Lean meat has a lower proportion of triacylglycerols and a higher proportion of phospholipids compared to meats with a higher intramuscular fat content and, consequently, contains proportionally more PUFAs, since they are preferentially deposited in muscle phospholipids [6]. In general, the ratio of omega‐6 FAs to *n*‐3 FAs in grazing‐finished cattle is more adequate due to the higher contribution of *n*‐3 FAs in these animals. In fresh meat, this study showed n‐3 and n‐6 ratio indices lower than those observed by Mottin et al. [38] in feedlot animals. TL levels in commercial cattle can range from 1% to 10% [39]. In this study, TL rates were lower than most of the values reported in the literature, as observed by Rossato et al. [39]; however, the results were higher than those observed by Mottin et al. [38].

A balanced *n*‐6/*n*‐3 ratio is an important dietary factor in the prevention of obesity, due to the pro‐ and anti‐inflammatory properties exerted by PUFAs themselves. Appropriate dietary intervention is important in the prevention and treatment of obesity, especially avoiding chronic/long‐term inflammatory states [40]. Inflammation is a predisposing factor to cancer, as *n*‐3 have anti‐inflammatory properties; it is important to include food sources of these FAs to preserve health. In our study, feedlot animals had an n‐6/n‐3 ratio in meat that was less favorable to human health compared to animals raised fully extensively, which is in line with other studies [15].

Total SFAs showed higher concentrations in the extensive native pasture systems, Santa Cruz and Monte Alegre, in both periods, which can be explained by the high values of C16:0 found in the pastures of these systems. The sum of MUFAs was higher in extensive grazing (Monte Alegre) in the dry season and in the feedlot in the rainy season, which can also be observed in the work of Silva et al. [15]. In this case, it is possible to infer that the feedlot system exerts an influence on MUFA levels. Bressan et al. [41] showed that the production system plays an important role in the composition of bovine FA. However, these authors reported that *B. indicus* animals have a higher percentage of SFAs and a lower percentage of MUFAs than *B. taurus* animals finished in a feedlot. According to them, *B. indicus* cattle finished in a feedlot have a lower capacity to desaturate SFAs. BCFAs are mainly SFAs with one or more methyl branches in the carbon chain.

As for the *h/H* ratio, it relates the functional activity of FA in relation to aspects of the metabolism of plasma cholesterol‐transporting lipoproteins, the quantification of which reflects the greater or lesser risk of incidence of cardiovascular diseases [42]. For meat products, a value of 2 is considered, which represents the ideal ratio between hyper‐ and hypocholesterolemic FAs [15]. In this case, the values observed in intensive grazing in the rainy season and in the feedlot in the dry season correspond to fats of superior nutritional quality.

The highest values found in AI, in extensive grazing (Monte Alegre) and in the feedlot, and in the TI, in extensive grazing (Monte Alegre) in the rainy season, represent the FA that can prevent the onset of atherosclerosis and coronary thrombosis, based on its effects on serum cholesterol and LDL cholesterol concentrations. Because the lower the values of AI and TI, the greater the amount of antiatherogenic FA present [22]. In this case, meats with low TI and AI values can be considered of better nutritional quality for humans [43]. The higher values found in the AI of the muscle may have occurred mainly because they have higher concentrations of C16:0 and myristic acid (C14:0), which are more atherogenic and have lower concentrations of PUFAs.

The animals in the feedlot system showed a tendency of superiority for the activity of the enzyme Δ9‐desaturase16, responsible for the transformation of C16:0 into palmitoleic acid, and Δ9‐desaturase18, which transforms stearic acid into oleic acid. The activity index of the enzyme Δ9 desaturase18 is calculated by considering the levels of oleic acid in the tissue. In this case, the higher the activity of the enzyme, the greater the synthesis of oleic acid, which can be seen in the results [44]. The enzyme elongase showed higher activity in animals from the extensive grazing (Santa Cruz) in both seasons and in feedlot systems in the dry season. Elongase has important metabolic functions, such as the formation of long‐chain PUFAs [35]. The results observed in the present study were superior to those found by Lopes et al. [27].

Due to the growing aversion to fat visible in retail and due to consumers’ concern about cardiovascular diseases, muscle can be an important source of protein and higher concentrations of *n*‐6 and *n*‐3 FA, whose importance for human nutrition is recognized, since the body does not have the efficient capacity to synthesize them [45]. It is important to emphasize that meat is not the only ingredient in the human diet and that one should consider not only its triglyceride content, but the total content of the diet consumed. The consumption of beef should be encouraged, as it contains FAs that are beneficial to human health, such as C18:1*c*9 and CLA, which are later found only in animal products, especially ruminants [27].

Another factor that could influence the results is genetics, since, despite being Zebu animals, all of them from the Nelore breed, the latter has different bloodlines [45]. However, the animals used were purchased from the same producer, where the breeding lineage is standardized, so the genetic factor possibly did not influence the results.

The different practices used on farms characterize the farming systems in the Eastern Amazon. Farming systems are adaptations made due to soil and climate characteristics, and it is precisely these factors that influence tissue composition. Our work has shown that the strategies used in each system, even when using animals of the same origin, influence the animals’ tissue composition, as the systems have different particularities.

## 5. Conclusion

The FA composition of the analyzed animals was mainly influenced by the rearing systems. In this case, the study showed that pasture production, such as traditional extensive systems, promoted a better proportion of *n*‐6/*n*‐3, contributing to a healthier lipid composition in the meat, when compared to feedlot. Although the feedlot system has a lower concentration of SFA, the analysis shows that the extensive system offers a more balanced and healthy FA composition.

## Author Contributions

Conceptualization: José de Brito Lourenço Júnior, André Guimarães Maciel e Silva, and Jamile Andréa Rodrigues da Silva; methodology: Andrea Viana da Cruz, Ana Paula Damasceno Ferreira, Adriny dos Santos Miranda Lobato, and Thomaz Cyro Guimarães de Carvalho Rodrigues; software: Andrea Viana da Cruz and Susana Paula Alves; formal analysis: Andrea Viana da Cruz, Ana Paula Damasceno Ferreira, Adriny dos Santos Miranda Lobato, Cristina Fernandes Xavier, and Rui José Branquinho Bessa; resources: José de Brito Lourenço Júnior; investigation: all authors; data curation: Andrea Viana da Cruz, Susana Paula Alves, and Elton Alex Corrêa da Silva; writing–original draft preparation: Andrea Viana da Cruz, Susana Paula Alves, Leonel António Joaquim, Laíza de Kássia Mendes da Conceição, Mariana Jucá Moraes, and Welligton Conceição da Silva; writing–review and editing: Welligton Conceição da Silva, Vanessa Vieira Lourenço Costa, and José de Brito Lourenço Júnior; supervision: José de Brito Lourenço Júnior, Jamile Andréa Rodrigues da Silva, and Susana Paula Alves; project administration: José de Brito Lourenço Júnior, André Guimarães Maciel e Silva, Jamile Andréa Rodrigues da Silva, and Antônio Marcos Quadros Cunha; and funding acquisition: José de Brito Lourenço Júnior.

## Funding

This research was supported by the Centro Interdisciplinar de Investigação em Sanidade Animal, Grant No. UIDB/00276/2020, Associated Laboratory for Animal and Veterinary Science, Grant No. LA/P/0059/2020), and LEAF, Grant No. UIDB/04129/2020.

## Disclosure

All authors have read and agreed to the published version of the manuscript.

## Conflicts of Interest

The authors declare no conflicts of interest.

## Supporting Information

Additional supporting information can be found online in the Supporting Information section.

## Supporting information


**Supporting Information** Table S1: Mean concentrations (% total identified peaks) and estimated marginal means ± standard errors of 52 FAs and dimethyl acetals (DMAs) in the *Longissimus lumborum* muscle of Nelore cattle.

## Data Availability

The data that support the findings of this study are available from the corresponding author upon reasonable request.
