# Supplementary material for: Effect of Rearing System and Seasonality on Fatty Acid Composition in Longissimus lumborum Muscle of Cattle in Eastern Amazon, Brazil
Source: Vet Med Int. 2026 Aug 25;2026:2468294. doi: 10.1155/vmi/2468294 (PMC13505193; doi:10.1155/vmi/2468294)
Supplement: Supplementary file 1 [file VMI-2026-2468294-s001.docx]

**Table S1.** Mean concentrations (% total identified peaks) and estimated marginal means ± standard errors.of 52 fatty acids and dimethyl acetals (DMA) in the *Longissimus lumborum* muscle of Nelore cattle.

|  | Extensive grazing | | | | São Miguel (Intensive grazing) | | Feedlot (Santa Izabel) | | **Systems** | **Season** | **Systems x Season** |
| --- | --- | --- | --- | --- | --- | --- | --- | --- | --- | --- | --- |
|  | Santa Cruz | | Monte Alegre | |  |  |  |  |  |  |  |
|  | **RS** | **DS** | **RS** | **DS** | **RS** | **DS** | **RS** | **DS** |  |  |  |
| Saturated and branched-chain | |  |  |  |  |  |  |  |  |  |  |
| C10_0 | 0.05 ± 0.01 aB | 0.07 ± 0.01 aA | 0.06 ± 0.01 aA | 0.06 ± 0.01 aA | 0.02 ± 0.01 bA | 0.04 ± 0.01 bA | 0.05 ± 0.0043 aA | 0.02 ± 0.0043 bB | P < 0,001 | 0,500 | P < 0,001 |
| C12_0 | 0.07 ± 0.01 aA | 0.09 ± 0.01 bA | 0.07 ± 0.0041 aA | 0.08 ± 0.0041 bA | 0.07 ± 0.02 aB | 0.23 ± 0.02 aA | 0.07 ± 0.01 aA | 0.04 ± 0.01 cB | P < 0,001 | P < 0,001 | P < 0,001 |
| i_C14_0 | 0.18 ± 0.03 aA | 0.14 ± 0.03 aA | 0.10 ± 0.01 bA | 0.11 ± 0.01 aA | 0.05 ± 0.01 cB | 0.08 ± 0.01 aA | 0.04 ± 0.01 cA | 0.02 ± 0.01 bA | P < 0,001 | 0,685 | 0,011 |
| C14:0 | 1.89 ± 0.19 bA | 1.99 ± 0.19 bA | 2.86 ± 0.12 aA | 2.33 ± 0.12 bB | 1.58 ± 0.25 bB | 3.06 ± 0.25 aA | 3.15 ± 0.21 aA | 1.75 ± 0.21 bB | P < 0,001 | 0,536 | P < 0,001 |
| i-C15:0 | 0.30 ± 0.02 aA | 0.25 ± 0.02 aA | 0.31 ± 0.02 aA | 0.29 ± 0.02 aA | 0.11 ± 0.01 bB | 0.16 ± 0.01 bA | 0.11 ± 0.02 bA | 0.05 ± 0.02 cB | P < 0,001 | 0,103 | P < 0,001 |
| a-C15:0 | 0.36 ± 0.04 aA | 0.35 ± 0.04 aA | 0.32 ± 0.03 aA | 0.31 ± 0.03 aA | 0.14 ± 0.01 bB | 0.22 ± 0.01 bA | 0.16 ± 0.03 bA | 0.12 ± 0.03 cA | P < 0,001 | 0,788 | 0,012 |
| C15:0 | 0.48 ± 0.04 aB | 0.61 ± 0.04 aA | 0.44 ± 0.03 aA | 0.44 ± 0.03 bA | 0.24 ± 0.01 bB | 0.36 ± 0.01 bcA | 0.38 ± 0.03 aA | 0.29 ± 0.03 cA | P < 0,001 | 0,069 | P < 0,001 |
| i-C16:0 | 0.28 ± 0.01 aA | 0.27 ± 0.01 aA | 0.27 ± 0.02 aA | 0.27 ± 0.02 aA | 0.12 ± 0.02 bB | 0.21 ± 0.02 bA | 0.14 ± 0.02 bA | 0.12 ± 0.02 cA | P < 0,001 | 0,189 | 0,003 |
| C16:0 | 21.06 ± 0.87 cA | 22.34 ± 0.87 aA | 24.99 ± 0.48 aA | 23.99 ± 0.48 aA | 21.68 ± 0.66 bcB | 23.95 ± 0.66 aA | 24.28 ± 0.75 abA | 18.51 ± 0.75 bB | P < 0,001 | 0,107 | P < 0,001 |
| i-C17:0 | 0.45 ± 0.02 aA | 0.42 ± 0.02 aA | 0.44 ± 0.02 aA | 0.45 ± 0.02 aA | 0.26 ± 0.01 bB | 0.31 ± 0.01 bA | 0.24 ± 0.02 bA | 0.27 ± 0.02 bA | P < 0,001 | 0,181 | 0,045 |
| a-C17:0 | 0.57 ± 0.03 bA | 0.58 ± 0.03 bA | 0.69 ± 0.03 aA | 0.69 ± 0.03 aA | 0.26 ± 0.02 dB | 0.44 ± 0.02 cA | 0.41 ± 0.02 cA | 0.38 ± 0.02 cA | P < 0,001 | 0,018 | P < 0,001 |
| C17:0 | 1.13 ± 0.04 aA | 1.24 ± 0.04 aA | 1.08 ± 0.05 aA | 1.10 ± 0.05 aA | 0.45 ± 0.04 cB | 0.71 ± 0.04 bA | 0.77 ± 0.05 bA | 0.80 ± 0.05 bA | P < 0,001 | 0,001 | 0,016 |
| i-C18:0 | 0.27 ± 0.02 aA | 0.28 ± 0.02 aA | 0.25 ± 0.01 aA | 0.25 ± 0.01 aA | 0.07 ± 0.01 bB | 0.13 ± 0.01 bA | 0.11 ± 0.01 bA | 0.07 ± 0.01 cB | P < 0,001 | 0,493 | 0,011 |
| C18:0 | 24.96 ± 0.98 a | 23.39 ± 0.98 a | 20.40 ± 0.85 b | 20.13 ± 0.85 b | 17.39 ± 0.63 c | 18.87 ± 0.63 c | 15.42 ± 0.97 c | 18.46 ± 0.97 c | P < 0,001 | 0,275 | 0,073 |
| C20:0 | 0.22 ± 0.02 aA | 0.19 ± 0.02 aA | 0.14 ± 0.01 bA | 0.14 ± 0.01 aA | 0.11 ± 0.01 bcB | 0.14 ± 0.01 aA | 0.09 ± 0.01 cB | 0.15 ± 0.01 aA | P < 0,001 | 0,088 | 0,003 |
| C22:0 | 0.01 ± 0.01 bA | 0.01 ± 0.01 bA | 0.01 ± 0.01 bA | 0.01 ± 0.01 bA | 0.09 ± 0.01 aA | 0.01 ± 0.01 bB | 0.04 ± 0.01 bA | 0.07 ± 0.01 aA | P < 0,001 | 0,060 | P < 0,001 |
| Monounsaturated (FA) | |  |  |  |  |  |  |  |  |  |  |
| C14:1c9 | 0.25 ± 0.03 cA | 0.26 ± 0.03 bA | 0.38 ± 0.02 bA | 0.34 ± 0.02 bA | 0.26 ± 0.06 bcB | 0.52 ± 0.06 aA | 0.84 ± 0.07 aA | 0.35 ± 0.07 abB | P < 0,001 | 0,052 | P < 0,001 |
| C16:1c7 | 0.26 ± 0.01 aB | 0.31 ± 0.01 aA | 0.23 ± 0.01 bB | 0.23 ± 0.01 bA | 0.19 ± 0.01 abB | 0.23 ± 0.01 abA | 0.15 ± 0.02 cB | 0.20 ± 0.02 cA | P < 0,001 | 0,001 | 0,226 |
| C16:1c9 | 1.57 ± 0.09 bA | 1.69 ± 0.09 bA | 1.90 ± 0.10 bA | 1.83 ± 0.10 abA | 1.60 ± 0.11 bB | 2.13 ± 0.11 aA | 2.78 ± 0.16 aA | 1.83 ± 0.16 abB | P < 0,001 | 0,267 | P < 0,001 |
| C17:1c9 | 0.66 ± 0.03 a | 0.70 ± 0.03 a | 0.60 ± 0.02 b | 0.62 ± 0.02 b | 0.34 ± 0.05 c | 0.44 ± 0.05 c | 0.61 ± 0.06 ab | 0.67 ± 0.06 ab | P < 0,001 | 0,053 | 0,580 |
| C18:1t6/7/8 | 0.18 ± 0.01 b | 0.19 ± 0.01 b | 0.14 ± 0.01 c | 0.14 ± 0.01 c | 0.32 ± 0.04 a | 0.35 ± 0.04 a | 0.18 ± 0.01 b | 0.16 ± 0.01 bA | P < 0,001 | 0,659 | 0,679 |
| C18:1t9 | 0.21 ± 0.01 abA | 0.22 ± 0.01 abA | 0.17 ± 0.01 bA | 0.19 ± 0.01 bcA | 0.26 ± 0.03 aA | 0.29 ± 0.03 aA | 0.20 ± 0.01 abA | 0.16 ± 0.01 cB | P < 0,001 | 0,386 | 0,005 |
| C18:1t10 | 0.22 ± 0.03 bA | 0.21 ± 0.03 cA | 0.22 ± 0.03 bA | 0.20 ± 0.03 cA | 0.28 ± 0.03 bB | 0.54 ± 0.03 bA | 0.57 ± 0.06 aB | 0.78 ± 0.06 aA | P < 0,001 | P < 0,001 | P < 0,001 |
| C18:1t11 | 2.06 ± 0.14 aB | 2.50 ± 0.14 aA | 1.50 ± 0.12 bA | 1.56 ± 0.12 bA | 1.13 ± 0.10 bcB | 1.48 ± 0.10 bA | 0.89 ± 0.11 cA | 0.43 ± 0.11 cB | P < 0,001 | 0,269 | P < 0,001 |
| C18:1t12 | 0.17 ± 0.02 bA | 0.08 ± 0.02 bB | 0.04 ± 0.01 cA | 0.05 ± 0.01 bA | 0.36 ± 0.04 aA | 0.25 ± 0.04 aB | 0.09 ± 0.03 cB | 0.17 ± 0.03 aA | P < 0,001 | 0,103 | P < 0,001 |
| C18:1c9 | 30.81 ± 0.73 bcA | 32.67 ± 0.73 abA | 34.73 ± 1.32 abA | 35.73 ± 1.32 aA | 28.65 ± 0.77 cB | 32.35 ± 0.77 abA | 37.15 ± 1.24 aA | 29.93 ± 1.24 bB | P < 0,001 | 0,828 | P < 0,001 |
| C18:1c11 | 0.87 ± 0.06 bA | 0.86 ± 0.06 cA | 0.96 ± 0.06 abA | 1.12 ± 0.06 bA | 1.16 ± 0.06 aA | 1.16 ± 0.06 bA | 1.18 ± 0.08 aB | 1.89 ± 0.08 aA | P < 0,001 | P < 0,001 | P < 0,001 |
| C18:1c12 | 0.11 ± 0.02 abA | 0.09 ± 0.02 cA | 0.08 ± 0.01 bA | 0.10 ± 0.01 cA | 0.10 ± 0.01 abB | 0.19 ± 0.01 bA | 0.17 ± 0.03 aB | 0.31 ± 0.03 aA | P < 0,001 | P < 0,001 | P < 0,001 |
| C18:1c13 | 0.13 ± 0.02 bA | 0.08 ± 0.02 bA | 0.15 ± 0.02 bA | 0.18 ± 0.02 aA | 0.08 ± 0.02 bB | 0.15 ± 0.02 abA | 0.28 ± 0.03 aA | 0.19 ± 0.03 aB | P < 0,001 | 0,583 | P < 0,001 |
| C18:1t16/c14 | 0.19 ± 0.02 b | 0.20 ± 0.02 b | 0.16 ± 0.01 b | 0.17 ± 0.01 b | 0.21 ± 0.01 a | 0.25 ± 0.01 a | 0.17 ± 0.01 b | 0.16 ± 0.01 b | P < 0,001 | 0,255 | 0,322 |
| C18:1c15 | 0.09 ± 0.02 aA | 0.08 ± 0.02 abA | 0.08 ± 0.01 aA | 0.10 ± 0.01 aA | 0.05 ± 0.01 aB | 0.10 ± 0.01 aA | 0.07 ± 0.01 aA | 0.04 ± 0.01 bB | P < 0,001 | 0,482 | P < 0,001 |
| C18:1c16 | 0.02 ± 0.0049 bA | 0.02 ± 0.0049 aA | 0.02 ± 0.0044 bA | 0.02 ± 0.0044 aA | 0.04 ± 0.01 aA | 0.02 ± 0.01 aB | 0.03 ± 0.0022 abA | 0.0014 ± 0.0022 bB | 0,005 | P < 0,001 | P < 0,001 |
| C19:1c9 | 0.17 ± 0.01 aA | 0.17 ± 0.01 aA | 0.18 ± 0.01 aA | 0.16 ± 0.01 aA | 0.07 ± 0.02 bA | 0.11 ± 0.02 bA | 0.14 ± 0.02 aA | 0.08 ± 0.02 bB | P < 0,001 | 0,193 | 0,040 |
| C19:1c11 | 0.03 ± 0.01 b | 0.02 ± 0.01 b | 0.04 ± 0.01 a | 0.05 ± 0.01 a | 0.01 ± 0.01 b | 0.02 ± 0.01 b | 0.03 ± 0.01 ab | 0.04 ± 0.01 ab | P < 0,001 | 0,244 | 0,790 |
| C20:1 | 0.15 ± 0.01 aA | 0.14 ± 0.01 bA | 0.12 ± 0.01 aB | 0.18 ± 0.01 abA | 0.11 ± 0.01 aB | 0.17 ± 0.01 abA | 0.13 ± 0.01 aB | 0.19 ± 0.01 aA | 0,357 | P < 0,001 | 0,002 |
| CLA-c9t11 | 0.42 ± 0.02 aB | 0.55 ± 0.02 aA | 0.35 ± 0.02 abA | 0.40 ± 0.02 bA | 0.29 ± 0.02 bB | 0.41 ± 0.02 bA | 0.33 ± 0.02 bA | 0.18 ± 0.02 cB | P < 0,001 | 0,009 | P < 0,001 |
| Polyunsaturated (FA) | |  |  |  |  |  |  |  |  |  |  |
| C18:2tt | 0.07 ± 0.01 bA | 0.06 ± 0.01 aA | 0.07 ± 0.01 bA | 0.08 ± 0.01 aA | 0.09 ± 0.02 abA | 0.07 ± 0.02 aA | 0.13 ± 0.01 aA | 0.02 ± 0.01 bB | 0,680 | P < 0,001 | P < 0,001 |
| C18:2c9t15/t8c13 | 0.02 ± 0.01 bA | 0.03 ± 0.01 aA | 0.05 ± 0.01 aA | 0.04 ± 0.01 aA | 0.04 ± 0.01 abA | 0.05 ± 0.01 aA | 0.07 ± 0.0033 aA | 0.0028 ± 0.0033 bB | 0,016 | 0,002 | P < 0,001 |
| C18:2t9c12 | 0.03 ± 0.01 abA | 0.04 ± 0.01 aA | 0.02 ± 0.0035 abA | 0.03 ± 0.0035 aA | 0.04 ± 0.01 aA | 0.02 ± 0.01 aA | 0.02 ± 0.0016 bA | 0. ± 0.0016 bB | P < 0,001 | 0,123 | P < 0,001 |
| C18:2c9t12 | 0.02 ± 0.01 aA | 0.01 ± 0.01 abA | 0.04 ± 0.01 aA | 0.01 ± 0.01 abB | 0.03 ± 0.01 aA | 0.02 ± 0.01 aA | 0.04 ± 0.0024 aA | 0.0028 ± 0.0024 bB | 0,504 | P < 0,001 | P < 0,001 |
| C18:2t11c15 | 0.11 ± 0.02 aA | 0.15 ± 0.02 aA | 0.13 ± 0.01 aA | 0.14 ± 0.01 abA | 0.05 ± 0.01 bB | 0.10 ± 0.01 bA | 0.05 ± 0.01 bA | 0.0021 ± 0.01 cB | P < 0,001 | 0,130 | P < 0,001 |
| C18:2n-6 | 3.54 ± 0.42 bA | 2.81 ± 0.42 cA | 2.10 ± 0.21 cA | 2.49 ± 0.21 cA | 12.09 ± 0.82 aA | 5.80 ± 0.82 bB | 5.28 ± 0.97 bB | 13.35 ± 0.97 aA | P < 0,001 | 0,451 | P < 0,001 |
| C18:3n-6 | 0.01 ± 0.0034 bA | 0.01 ± 0.0034 bA | 0.01 ± 0.0043 bA | 0.02 ± 0.0043 bA | 0.04 ± 0.0035 aA | 0.0029 ± 0.0035 bB | 0.02 ± 0.01 abA | 0.05 ± 0.01 aA | P < 0,001 | 0,741 | P < 0,001 |
| C18:3n-3 | 1.02 ± 0.09 bA | 0.79 ± 0.09 aA | 0.75 ± 0.06 bcA | 0.80 ± 0.06 aA | 1.70 ± 0.10 aA | 0.68 ± 0.10 aB | 0.46 ± 0.13 cB | 0.89 ± 0.13 aA | P < 0,001 | 0,005 | P < 0,001 |
| C20:2n-6 | 0.03 ± 0.01 bA | 0.03 ± 0.01 bcA | 0.02 ± 0.0033 bA | 0.03 ± 0.0033 cA | 0.07 ± 0.01 aA | 0.05 ± 0.01 bA | 0.05 ± 0.02 abB | 0.15 ± 0.02 aA | P < 0,001 | P < 0,001 | P < 0,001 |
| C20:3n-9 / 18:3 c9t11c15 | 0.32 ± 0.05 aA | 0.17 ± 0.05 abB | 0.14 ± 0.01 bA | 0.15 ± 0.01 bA | 0.25 ± 0.02 aA | 0.18 ± 0.02 abB | 0.11 ± 0.02 bB | 0.24 ± 0.02 aA | P < 0,001 | 0,379 | P < 0,001 |
| C20:3n-6 | 0.30 ± 0.03 bA | 0.29 ± 0.03 bA | 0.17 ± 0.02 cA | 0.20 ± 0.02 bA | 0.55 ± 0.04 aA | 0.28 ± 0.04 bB | 0.22 ± 0.07 bcB | 0.65 ± 0.07 aA | P < 0,001 | 0,176 | P < 0,001 |
| C20:4n-6 | 1.33 ± 0.13 bA | 0.92 ± 0.13 bB | 0.60 ± 0.08 cA | 0.72 ± 0.08 bA | 2.62 ± 0.24 aA | 1.11 ± 0.24 bB | 0.94 ± 0.22 bcB | 2.61 ± 0.22 aA | P < 0,001 | 0,792 | P < 0,001 |
| C20:5n-3 | 0.47 ± 0.06 aA | 0.28 ± 0.06 bB | 0.17 ± 0.02 bB | 0.23 ± 0.02 bA | 0.70 ± 0.08 aA | 0.32 ± 0.08 bB | 0.19 ± 0.08 bB | 0.65 ± 0.08 aA | P < 0,001 | 0,800 | P < 0,001 |
| C22:4n-6 | 0.14 ± 0.03 abA | 0.11 ± 0.03 bA | 0.04 ± 0.01 cA | 0.07 ± 0.01 bA | 0.18 ± 0.02 aA | 0.10 ± 0.02 bB | 0.10 ± 0.02 bcB | 0.23 ± 0.02 aA | P < 0,001 | 0,432 | P < 0,001 |
| C22:5n-3 | 0.86 ± 0.07 bA | 0.64 ± 0.07 bB | 0.41 ± 0.05 cA | 0.47 ± 0.05 bA | 1.23 ± 0.12 aA | 0.67 ± 0.12 bB | 0.44 ± 0.12 cB | 1.18 ± 0.12 aA | P < 0,001 | 0,903 | P < 0,001 |
| C22:6n-3 | 0.04 ± 0.02 abA | 0.04 ± 0.02 abA | 0.03 ± 0.01 bA | 0.03 ± 0.01 bA | 0.10 ± 0.01 aA | 0.04 ± 0.01 bB | 0.03 ± 0.01 bB | 0.10 ± 0.01 aA | P < 0,001 | 0,805 | P < 0,001 |
| Dimethyl acetals (DMA) | |  |  |  |  |  |  |  |  |  |  |
| DMA-C16:0 | 0.43 ± 0.07 bA | 0.17 ± 0.07 bB | 0.45 ± 0.07 bA | 0.28 ± 0.07 abA | 1.13 ± 0.21 aA | 0.23 ± 0.21 abB | 0.19 ± 0.07 bB | 0.44 ± 0.07 aA | 0,088 | 0,002 | P < 0,001 |
| DMA-C18:0 | 0.40 ± 0.06 abA | 0.16 ± 0.06 bB | 0.33 ± 0.06 bA | 0.20 ± 0.06 bA | 0.84 ± 0.16 aA | 0.20 ± 0.16 abB | 0.19 ± 0.07 bB | 0.49 ± 0.07 aA | 0,158 | 0,011 | P < 0,001 |
| DMA-C18:1 | 0.01 ± 0.01 bA | 0.01 ± 0.01 aA | 0.0011 ± 0.0032 bB | 0.01 ± 0.0032 aA | 0.10 ± 0.03 aA | 0.01 ± 0.03 aB | 0.02 ± 0.01 bA | 0.03 ± 0.01 aA | P < 0,001 | 0,048 | 0,048 |

When a significant interaction was detected, different lowercase letters within the same row indicate significant differences among rearing systems within each season, whereas different uppercase letters within the same row indicate significant differences between seasons within each rearing system (Sidak adjustment, P < 0.05). When the interaction was not significant, lowercase and uppercase letters indicate differences among the main effects evaluated separately (Sidak adjustment, P < 0.05). P-values for rearing system, season, and their interaction were obtained using Type III Wald chi-square tests. DS = dry season; DR = rainy season.
